# Virtual self-conversation using motivational interviewing techniques to promote healthy eating and physical activity: A usability study

**DOI:** 10.3389/fpsyt.2023.999656

**Published:** 2023-04-19

**Authors:** Dimitra Anastasiadou, Pol Herrero, Julia Vázquez-De Sebastián, Paula Garcia-Royo, Bernhard Spanlang, Elena Álvarez de la Campa, Mel Slater, Andreea Ciudin, Marta Comas, J. Antoni Ramos-Quiroga, Pilar Lusilla-Palacios

**Affiliations:** ^1^Psychiatry, Mental Health and Addictions Research Group, Vall d’Hebron Research Institute, Barcelona, Spain; ^2^Department of Clinical and Health Psychology, Autonomous University of Barcelona, Barcelona, Spain; ^3^RE-FiT Barcelona Research Group, Vall d’Hebron Research Institute and Parc Sanitari Pere Virgili, Barcelona, Spain; ^4^Virtual Bodyworks S.L., Barcelona, Spain; ^5^The Institute of Neurosciences of the University of Barcelona, Barcelona, Spain; ^6^Department of Clinical Psychology and Psychobiology, University of Barcelona, Barcelona, Spain; ^7^Endocrinology and Nutrition Department, Vall d’Hebron University Hospital, Barcelona, Spain; ^8^Vall d’Hebron Research Institute (VHIR), Universitat Autònoma de Barcelona (VHIR-UAB), Barcelona, Spain; ^9^Centro de Investigación Biomédica en Red de Diabetes y Enfermedades Metabólicas Asociadas (CIBERDEM), Instituto de Salud Carlos III, Madrid, Spain; ^10^Psychiatry Department, Vall d’Hebron University Hospital, Barcelona, Spain; ^11^Department of Psychiatry and Legal Medicine, Universitat Autònoma de Barcelona, Barcelona, Spain; ^12^Biomedical Network Research Centre on Mental Health (CIBERSAM), Barcelona, Spain

**Keywords:** obesity, virtual reality, psychological treatment, embodiment, usability, motivational interviewing

## Abstract

**Introduction:**

We aim to examine the usability of a Virtual Reality (VR) platform, called *ConVRSelf*, which has been designed to address the needs of People Living With Obesity (PLWO).

**Methods:**

Fourteen participants with a desire to eat healthier and exercise more (6 normal weight and 8 PLWO; Mean age = 41.86, *SD* = 13.89) were assigned to the experimental group (EG) or the control group (CG). EG participants, after being trained on motivational interviewing skills, engaged in a virtual self-conversation using embodiment and body swapping techniques, which aimed to normalize and resolve their ambivalence to change lifestyle habits. CG participants, embodied in their virtual bodies, participated in a pre-established discourse with a virtual counselor giving them psychoeducational advice about how to change lifestyle habits. A mixed-methods design was used, involving a semi-structured interview and self-report questionnaires, including readiness to change habits (Readiness Rulers), body ownership (Body Ownership Questionnaire, BOQ), and system usability (System Evaluation Questionnaire, SEQ). Thematic content analysis was carried out for qualitative data while statistical data analysis was carried out using SPSS 20.0.

**Results:**

Participants from both groups showed high readiness to change lifestyle (Readiness Rulers) before engaging with the virtual experiences, which was maintained at the same level after the interventions and their scores on the SEQ and BOQ were satisfactory. Regarding qualitative information obtained from the interviews, almost all participants found the VR experience to be novel, interesting, and enjoyable. A higher acceptability was observed among PLWO from the EG than normal weight participants from the same group, a promising finding for the *ConVRSelf* platform, which had been specifically designed to address the needs of PLWO.

**Conclusion:**

The *ConVRSelf* system is well-accepted by participants and is ready to be tested with PLWO in a clinical setting.

## Introduction

Obesity is a complex and chronic disease with multiple adverse effects on the individual’s physical and psychological health (1). Guidelines support psychological and behavioral weight management interventions as gold standard treatments for obesity (2). However, these treatments often fail in the long-term; in fact, nearly half of patients return to their original weight within 5 years of treatment (3). One of the factors associated with this may be the low attention paid in therapy to the motivating factors underlying adherence (4). Another factor may be the limited addressment of the weight bias internalization, often produced among this population, which further contributes to increased morbidity and mortality independently of Body Mass Index (BMI) (5).

In recent decades, the low adherence to obesity treatment has been addressed by using Motivational Interviewing (MI) techniques (6, 7). MI can be understood as *“a collaborative, goal-oriented method of communication between patient and healthcare professional with particular attention to the language of change. It is designed to strengthen an individual’s motivation for and movement toward a specific goal by eliciting and exploring the person’s own reasons for change within an atmosphere of acceptance and compassion*” (8). Research has shown that it is feasible to integrate MI techniques with behavioral and psychological interventions for obesity and that these interventions have the potential to improve health-related outcomes, especially in the long term (9).

Hence, following the MI theoretical framework, psychological treatments for obesity should normalize the ambivalence that People Living With Obesity (PLWO) often experience when starting their change process toward a healthier lifestyle, and then help them to explore their own reasons for change in line with their needs and values. Finally, psychological treatments should also promote the self-efficacy of this population, which may be hindered due to the increased weight bias internalization, among other factors (5, 10).

Besides, COVID-19 has shown us the need for personalized treatments, adapted to unforeseen circumstances and special contexts, and has been a tipping point for the implementation of eHealth interventions (11), also for the treatment of obesity (10). In particular, the use of Virtual Reality (VR) with embodiment techniques following Cognitive Behavioral Therapy (CBT) principles has promising preliminary effectiveness in the treatment of eating disorders (ED) and obesity (12). However, some of the above-mentioned psychological factors associated with this condition (i.e., ambivalence to change, internalization of weight bias) have not been properly addressed in these treatments, which essentially deliver the same CBT in a VR environment.

The research reported in this paper is part of an overall attempt to respond to these challenges by integrating some elements of the MI theoretical framework (6) in a VR context to treat obesity. This takes place in the European project SOCRATES.

The study uses a VR platform, called *ConVRSelf* (13, 14), specifically developed for PLWO. One of the main advantages of using VR is that in such an environment, the person’s real body can be apparently substituted by a life-sized virtual body. Hence, when participants wear a Head-Mounted display and look down toward their bodies, they see a virtual body coincident in space with their real body. Also, through real-time tracking of their limbs, when they move their real body, the virtual body can be programmed to move correspondingly and synchronously. The technological means through which the person is endowed with a life-sized virtual body that apparently substitutes their real body is called “embodiment.” Particularly with the *ConVRSelf* platform, participants can see their virtual body that looks like their own body by looking directly down toward themselves or by seeing it reflected in a virtual mirror so that the mirror will reflect back the avatar they embody. The perceptual consequence of embodiment is “body ownership,” the perceptual illusion that participants can experience when they feel that the virtual body is their body even though they know that this is not the case. This type of body ownership was demonstrated in a classic paper where visual-tactile synchrony was used to incorporate a rubber hand into the body representation of participants through synchronous tactile stimulation of the seen rubber hand and the out-of-sight corresponding real hand (15). Whole-body ownership of a virtual body seen from a first-person perspective was demonstrated by video-based VR (16), and computer graphics-based VR (17), and it has been repeatedly demonstrated ever since (18–21). In addition, it has been shown that the avatar embodied does not have to look, necessarily, like the actual person for there to be a strong illusion of body ownership (21). Finally, a further consequence of such embodiment in bodies that are quite different from the person’s own body is that it can lead to changes in attitudes, behaviors, and cognition. For instance, the above-mentioned papers illustrate changes in implicit racial bias and also improvement in performance in a cognitive test when embodied as Albert Einstein.

The *ConVRSelf* platform employs embodiment and body swapping techniques for self-counseling. Participants, once being embodied in their look-alike avatar and having achieved a sense of body ownership, they found themselves in front of a counselor sitting across the table. First, participants explain their problem to the counselor, and once finished, they are embodied in the counselor’s body and can see and hear their own look-alike avatar explain the problem (this change of avatars is called the body swapping technique). In this case, while embodied as the counselor, if they look down toward themselves, they will see the counselor’s body, and similarly, this body will be reflected in a virtual mirror. Now, as counselor, they can respond to their look-alike avatar by making comments or suggestions about the problem. After this, they swap back to their original (self) body and can see and hear the counselor respond to their problem. Continuing in this way, they can continue a self-conversation from the two embodied perspectives—as themselves and as the counselor.

Virtual embodiment has been used extensively in orthopedic rehabilitation, pain reduction, ED and obesity (22–26), while embodiment and body swapping techniques have been shown to be effective in helping people solve personal problems through a virtual self-conversation (13, 14).

The objective of the present study is to examine the usability of the *ConVRself* platform among PLWO and normal weight participants. This platform has been specifically developed for PLWO to help them address the following challenges related to their condition: first, normalize and resolve their ambivalence to change, second, better understand and address the impact of weight bias internalization, and third, increase their self-efficacy by setting realistic goals in line with their own values. There were two groups of participants: the Experimental Group (EG) engaged with a virtual self-conversation using the embodiment and body swapping elements of *ConVRSelf,* whereas the Control Group (CG) listened to a virtual pre-established discourse with only the embodiment element. With the present usability study, we hypothesize that the VR platform in its 2 versions (embodiment + body swapping vs. only embodiment) would be well-accepted by participants from both groups, with higher levels of usability perceived by PLWO compared to normal weight participants. We also hypothesize that participants of the EG would increase more their motivation to enhance with healthy lifestyle habits through the motivational self-conversation compared to the CG, while the same levels of body ownership would be expected between groups. This is a preliminary study before the start of the SOCRATES clinical trial, which is running throughout 2022 and whose protocol has been published recently (27).

## Methods

### Design

This is a quasi-experimental between-groups design with a single factor and two treatment levels: EG vs. CG. There were two assessment time-points—at baseline (T0) and post-intervention (T1). A mixed-methods design was used, involving a semi-structured interview and self-report questionnaires.

### Procedure

Both participants from the EG and CG tried the Experiment 1 *“Embodied discussion about the problem and solutions”* of the ongoing SOCRATES Randomized Controlled Trial (RCT), in 2 different versions: the EG experienced a scenario with both the embodiment and body swapping elements integrated into the virtual experience, and the CG experienced only the embodiment element.

### Experimental group

Participants from this group were embodied alternatively in their look-alike avatar and in their counselor’s avatar. To create the avatars, participants’ whole-body photos, as well as information about their body weight and height, were required. For the counselor’s avatar, participants were able to choose among different avatar options that were sex-matched (young normal weight, old normal weight, young overweight, and old overweight). Then, participants engaged in a virtual self-conversation aiming at helping them understand their own motivation and ambivalence to eat healthier and exercise more. By using the body swapping technique, participants were embodied alternately in their own virtual representation, who explained their problem to a virtual counselor, and in their counselor’s body, who tried to advice on this problem. The goal was for them to better understand their process of change, to be more compassionate, and more competent counselors for themselves during the virtual experience. To better guide this virtual self-conversation, participants from the EG received an intensive training on MI skills (6) before being exposed to the VR. The basic goal of the training was to help participants organize their future self-conversation so they would be able to guide themselves through a discourse of change based on their values and interests. More details about the contents of the training can be found in the protocol study (27) while the behavioral change techniques used in the intervention can be found in Table 1.

**Table 1:** Summary of the motivational interviewing micro-skills to elicit behavioral change^1^.

Open questions	These types of questions make the person reflect and elaborate on their discourse. In contrast to Closed questions, which usually have a short answer and the variety of answers is limited, Open questions help the counselor understand the person’s internal frame of reference given that the answers the patient can provide are richer.
Affirming	The objective of this micro-skill is to accentuate the positive parts of the discourse. To affirm is to clarify and acknowledge what is good. Affirmation also means to indicate the client’s strengths and intentions.
Reflecting	Reflective statements made by the counselor allow the patient to listen again to what he/she is expressing in different words to maintain a discourse of change. It must be necessarily selective, in that one chooses what to reflect from all the information the patient has provided. The main objective is to highlight what the counselor thinks is important to the patient.
Summarizing	They are understood as reflections that compile different kinds of information from the patients. Hence, patients not only listen to themselves describing their experiences, but they also hear reflected what they said in order to encourage change.
Elicit-Provide-Elicit	This is a sequence for information exchange that respects the client’s expertise and autonomy. Before giving any information to the patient, MI encourages to first acknowledge what is the patient’s knowledge, and also to ask for permission to give new information.
Decisional balance	This evaluation helps the client resolve ambivalence by weighing the pros and cons during the decision process. To do so, the patient has to provide his/her own reasons by giving equal exploration to both pros and cons for doing such a change.
Readiness Rulers	These are scales ranging from 1 to 10 that evaluate “importance,” “confidence,” and “readiness” to promote change. For “importance,” it is meant the perceived need the person has for change; for “confidence,” the evaluation is about the patient’s self-efficacy; and for “readiness,” the patient evaluates their preparation to mobilize toward change. For the Usability study, these variables are measured in terms of (a) achieving a healthy weight and (b) exercising more.

1 Miller W, Rollnick S. La entrevista motivacional: ayudar a las personas a cambiar. Barcelona: Paidós; 2015.

#### Control group

Participants from this group were embodied in their look-alike avatar, similarly to what has been previously described for the EG, and participated in a “pre-established discourse” provided by a counselor chosen by them (as previously described for the EG), who asked them about the perceived barriers for engagement with a healthier lifestyle, and gave pre-recorded practical recommendations about how to achieve a healthier and happier life, in terms of healthy eating and physical activity.

### Participants

Fourteen participants (6 normal weight and 8 PLWO; Mean age = 41.86, *SD* = 13.89), with a desire to make positive lifestyle changes in terms of eating healthier and exercising more, were recruited for the usability study from the Vall d’Hebron University Hospital, during the months of August and November 2021, after having read an informative leaflet about the SOCRATES project and having expressed their interest to be involved. Participants were assigned to one of the two treatment conditions. The EG comprised 3 normal weight participants and 5 PLWO, and the CG 3 normal weight participants and 3 PLWO. PLWO had BMI ≥ 30 kg/m^2^ and ≤45 kg/m^2^. Inclusion criteria for the whole sample were (a) age between 18 and 65 years, (b) minimal digital skills, (c) oral and written understanding of the Spanish language, and (d) agreement to sign the informed consent to participate, while exclusion criteria were (a) presence of non-stabilized severe mental disorder, (b) auditory or visual complications, (c) intellectual disability or any major illness seriously affecting cognitive performance, and (d) history of epilepsy. For the PLWO group, 2 additional exclusion criteria were established as follows: (a) BMI > 46 kg/m^2^, (b) not receiving ambulatory treatment at the Vall d’Hebron University Hospital, and (c) presence of an ED at least for 2 years. All inclusion and exclusion criteria were assessed through an initial clinical interview by clinical researchers of the team. More detailed information about this procedure can be found in the publication of the RCT protocol (27).

### Assessment measures

#### Sociodemographic and clinical variables

Information about participants’ age, biological sex, level of education, marital and employment status, clinical information including the presence of any physical or mental health problem, as well as the fulfillment of the inclusion criteria, were assessed at baseline (T0) through a semi-structured interview by researchers of the SOCRATES team at Vall d’Hebron Research Institute (VHIR). The weight of each participant was measured by trained staff, at the same time of the day for all participants, and using a digital scale while the height was informed by participants. Finally, BMI was calculated as weight in kilograms divided by height in square meters [W (kg)/H (m^2^)].

#### Readiness Rulers

Readiness Rulers (RR) (28) are Visual Analog Scales ranging from 1 to 10 that assess “importance,” “confidence,” and “readiness” to change. For the present study, only the Readiness to change variable was measured in terms of (a) achieving a healthy weight and (b) exercising more, before the virtual experiment (T0) and just after the experiment (T1) (28). Lower numbers represent “not prepared” to change, scores of 4–6 represent ambivalence in terms of “somewhat prepared to change,” and higher numbers represent “very prepared to change” or ongoing attempts of changing. The RR have been used in addiction studies showing good psychometric properties (29).

#### Suitability Evaluation Questionnaire

The Suitability Evaluation Questionnaire (SEQ) (30), which is a 14-item questionnaire designed to measure satisfaction, acceptance, and security of use in VR platforms, was administered at T1 (30). Thirteen questions of the SEQ are based on a 5-point Likert scale plus a last open-ended question offering participants the possibility to add comments if they wish. The total score of the questionnaire ranges from 13 (poor suitability) to 65 (excellent suitability). For the specific purposes of the present study, the word “rehabilitation” of Q11 was replaced by “obesity treatment.” Preliminary results from an initial attempt to validate the SEQ by Gil-Gómez and colleagues showed an acceptable internal consistency of the questionnaire (Cronbach’s alpha = 0.7) (30).

#### Body Ownership Questionnaire

At the end of the VR exposure (T1), participants completed a subjective rating of the illusion of body ownership through a 7-point Likert scale, where −3 meant “Strongly disagree” and + 3 “Strongly agree.” Questions were taken from a previous study evaluating *ConVRSelf* (14), and are the following: *“Even though the body I see might not physically look like me, I feel that the virtual body I see when I look down towards myself is my body”* (body ownership-look down)*; “Even though the body I see might not physically look like me, I feel that the virtual body I see reflected in the mirror is my body”* (body ownership-mirror)*; “I feel that the movements of the virtual body are caused by my own movements”* (body ownership-movements)*; “The body I see in the virtual world physically looks like me”* (self-recognition). For the EG only, the same questions were asked for both the participant’s embodiment on the self-side and the counselor’s side.

#### Users’ experience

A brief interview was carried out after the VR exposure (T1) to assess the satisfaction of participants with the VR experience and acceptability of the *ConVRSelf* tool, including questions related to their experience using the self-conversation technique, in a VR context, to cope with their condition.

### Data analysis

Statistical analyses of the data were carried out using SPSS 20.0 statistical software. Non-parametric tests were performed due to the small sample size. Descriptive data regarding participants’ sociodemographic and clinical characteristics, readiness to change, body ownership, and usability were calculated, and differences between groups (normal weight vs. PLWO and CG vs. EG) on each sociodemographic and clinical variable were assessed with a Chi-square or Mann-Whitney U test. A Wilcoxon signed-ranked test was used to examine changes in participants’ readiness to change (through RR) in terms of Exercise more and Lose weight, for the whole sample and each group separately (EG and CG). A Mann-Whitney U test was performed to explore differences between EG and CG on the SEQ and Body Ownership Questionnaire (BOQ). Additionally, for any sociodemographic result that was statistically significant for EG and CG, an ANOVA with Bootstrapping (1,000 samples) was performed with such for the variable as covariate for SEQ and BOQ. Finally, a Spearman’s correlation was used to analyze the relationship between participants’ BMI and SEQ total score. To obtain the value of the effect size for the Mann–Whitney U test we calculated the r effect size as $r=\frac{\left|Z\right|}{\sqrt{n}}$ . For ANOVA with bootstrapping, we interpreted eta squared (η^2^). According to Cohen criteria, r = 0.1/η^2^ = 0.01, r = 0.3/η^2^ = 0.06, and r = 0.5/η^2^ = 0.14 are small, medium, and large effect, respectively (31). Statistical significance was set at *p* < 0.05.

Thematic content analysis was performed to analyze the qualitative data collected from T1 interviews with the EG. First, members of the research team independently read the transcripts of the 14 interviews and sought to categorize the available information in terms of perceived advantages and disadvantages of the VR experience, separately for the EG and the CG. Categories followed a previous classification carried out by a member of the team (32). In case of disagreement between the assessors, consensus was achieved through discussion. No software or tool was used during this process. Translations from Spanish to English of all qualitative data from the interviews was carried out by a bilingual research assistant. Finally, the most representative examples for each theme for the EG were collected from the transcripts after discussion among researchers of the team.

## Results

### Sociodemographic information

The comparisons of sociodemographic and clinical characteristics between normal weight and PLWO participants are illustrated in Table 2. It is worth mentioning that the majority of normal weight participants (83.3%) had university degree studies, while the majority of PLWO (87.5%) were educated up to “Intermediate/high secondary education” level [*χ*^2^(2) = 10.60, *p* = 0.031]. Also, a Mann–Whitney U test reported significant differences in the BMI between normal weight participants and PLWO (*Z* = −3.10; *p* = 0.002), in Age (*Z* = −2.33; *p* = 0.020), and educational level [χ^2^(2) = 10.60, *p* = 0.031]. Same comparisons were also performed between EG and CG. No differences were shown between groups except for variable Sex [*N*_EG_ (Men = 1; Women = 7) *N*_CG_ (Men = 5; Women = 1)] [*χ*^2^(1) = 7.02, *p* = 0.008].

**Table 2:** Sociodemographic Characteristics of Participants at Baseline.

		Normal weight (*N* = 6)		PLWO (*N* = 8)				*Z; p*
		*M*	*SD*	*M*	*SD*			
**Age (years)**		51.1	13.3	34.9	10.1			–2.33; **0.02**
**Mean age of treatment onset (years)**		−	−	29.5	12.2	−		
**Treatment duration (months)**		−	−	6.1	3.7	−		
**BMI**		24.1	2.5	42.9	4.3			−3.10; **0.002**
		Normal weight (*N* = 6)		PLWO (*N* = 8)		Full sample (*N* = 14)		*X2; p*
		*N*	%	*N*	%	*N*	%	
**Sex**								0.22; .640
	Female	3	50	5	62.5	8	57.1	
	Male	3	50	3	37.5	6	42.8	
**Nationality**								0.73; .393
	Spanish	5	83.3	5	62.5	10	71.4	
	Latin-American	1	16.7	3	37.5	4	28.5	
**Marital Status**								3.28; .194
	Single	3	50	5	62.5	8	57.1	
	Married/partnered	3	50	3	37.5	6	42.8	
**Educational level**								10.60; **.031**
	Intermediate secondary education/vocational training	1	16.7	3	37.5	4	28.5	
	Intermediate/high secondary education	0	0	4	50	4	28.5	
	University or postgraduate	5	83.3	1	12.5	6	42.8	
**Employment**								6.12; .190
	Student	0	0	1	12.5	1	7.1	
	Part-time job	0	0	2	25	2	14.3	
	Full-time job	5	83.3	2	25	7	50	
	Sick leave	0	0	2	25	2	14.3	
	Retired	1	16.7	1	12.5	2	14.3	
**Physical illness**								2.43; .119
	Yes	2	33.3	6	75	8	57.1	
	No	4	66.6	2	25	6	42.8	
**Mental illness**								1.66; .198
	Yes	1	16.6	4	50	5	35.7	
	No	5	83.3	4	50	9	64.3	
**Physical symptoms**								-
	Epilepsy	1	16.7	0	0	1	7.1	
	Type 2 diabetes	1	16.7	0	0	1	7.1	
	Hypertension	0	0	1	12.5	1	7.1	
	Respiratory problems	0	0	2	25	2	14.3	
	Thyroid	1	16.7	1	12.5	2	14.3	
	Gastritis	0	0	1	12.5	1	7.1	
	Fatty liver disease	0	0	1	12.5	1	7.1	
	Choletithiasis	0	0	1	12.5	1	7.1	
	Sleep apnea	0	0	1	12.5	1	7.1	
**Mental symptoms**	Anxiety, Panic attacks	1	16.7	2	25	3	21.4	
	Depression	0	0	2	25	2	14.3	
	Insomnia	1	16.7	0	0	1	7.1	
	ADHD	0	0	1	12.5	1	7.1	

Bold values indicate statistically significant differences between healthy participants and PLWO. *M* = mean score, *SD* = standard deviation, BMI =Body mass index, ADHD = Attention deficit hyperactivity disorder.

**Table 3:** Perceived advantages and disadvantages of the VR experience by normal weight participants (NW) and patients (P) from the Experimental Group.

**ADVANTAGES**			
**Domain**	**Key themes**	**Examples of NW´ statements**	**Examples of P’ statements**
**Characteristics of the VR platform**	Perceived ease of use	*- “At the beginning I needed some instructions and guidance from the therapist, but then it was easy for me to use the platform”*	*- “The platform was very intuitive and easy to use”.* *- “It was easy to use but I would rather use it with my glasses on because it was blurry”*
	Perceived usefulness	*- “It confronts you with your goals and helps you to better organize your ideas”* *- “It helped me to reflect and be more aware of the steps I must follow to achieve my goals in a more concrete and tangible way”* *- “I have now set a day to take my first step towards the change I want to do”* *- “I would recommend the platform to a friend who wants to make any lifestyle change”* *- “I have been able to immerse myself into the virtual experience, gain confidence, and not feel ashamed when explaining my problem to someone I did not know”* *- “ConVRself can be a time- and cost-saving platform to be used as a complementary tool to face-to-face treatment”* *- “Sometimes it is better to train people in this way, rather than to take them to the real therapeutic office”*	*- “With this platform I have been able to give myself advice more easily than when I have a problem and I have to think how to solve it”* *- “It makes you think and say to yourself: “Wow, this is something I have to do”* *- “Sometimes, it is hard to listen to the others’ advice, and we tend to trust more our own beliefs. Contrary to this, this platform is very useful because it helps you listen to your own advices as if they were from the outside”* *- “I had the chance to put myself in the counselor’s shoes and talk to me the way I would like to be talked to”* *- “What I enjoyed the most is having the chance to make questions I would myself love to hear”* *- “I became fully aware of my overweight”* *- “Together with psychological and nutritional supervision, I think this platform could help me changing my lifestyle”* *- “It helped me a lot because I realized my problem has a solution”* *- “I would recommend this platform to my partner, who has some emotional problems.*
	Design and Personalization	*- “I found the virtual environment attractive and cosy, and at the same time, the fact that it was empty was relaxing and promoted my concentration”* *- “I liked the personalized avatars”* *- “I found the counselor really kind and empathetic"*	*- “The virtual environment was very relaxing”* *- “The avatar was very similar to me, it wore the same shirt and the face was as small as mine”* *- Very intuitive platform and very relaxing environment”* *- “I really like the avatar’s body and voices, and the environment was very relaxing”*
**DISADVANTAGES**			
**Domain**	**Key themes**	**Examples of NW’ statements**	**Examples of P´ statements**
**Characteristics of the VR platform**	Negative perception of ease of use	*- “It took a long time for the psychologist who was accompanying me during the experience to do the initial calibration”* *- “I would improve the sensitivity of the controllers, as it was difficult for me to use them properly and to press the buttons that I was asked to continue with the experience correctly”*	*- “I spent too much time trying to push the buttons to make the experience work as it should”* *- “Being left-handed was quite challenging because the platform thought I wanted to push the Exit button when I was just rising my right arm”*
	Negative perception of usefulness	*- “I felt impatient when having to listen to myself twice. I felt disconnected and I lost fluency in my argumentation”* *- “During the self-conversation, I felt disconnected and a bit stressed with the change of roles (being patient or counselor). I was lost and did not know if I had to talk as a patient or counselor”* *- “To use it properly, I consider it essential to receive previous training on the correct use of the platform and also on how to correctly formulate questions for the self-conversation”*	*- “It took me too much time to push the buttons. Sometimes I disconnected myself from the experience”*
	Problems with the design of the platform	*- “I would prefer a more realistic virtual environment”* *- “The self-avatar did not look like me; it was fatter than me, and this was annoying during the experience”* *- “Avatars were unrealistic. They looked like cartoons”*	*- “My avatar was different. It was slimmer and the chest was more compact”*
	Lack of personalization	*- “It would have been better to personalize the background of the virtual environment. For instance, I would have liked it if I could choose among different backgrounds, such as the therapist’s office or a beach. This could increase my introspection capacity”* *- “It would have been nice to be able to regulate the luminosity of the virtual environment according to each user’s preferences”*	
**Individual factors**	Age, gender, and education	*- “I think that elderly people will have more problems in using the platform”*	
**Characteristics of the Oculus Quest 2**	Negative perception of ease of use	*- “The HMD was heavy and uncomfortable to wear after a long time”* *- “I had to wait after the experience because the area around my eyes was red from the pressure of the device”* *- “My lenses were dirty, and I wonder whether they had been properly disinfected before I used them for Covid-19”*	

### Readiness to change

A Wilcoxon signed-rank test showed that the virtual experiment with *ConVRSelf* did not elicit a statistically significant change in Readiness to exercise more (*Z* = −0.74, *p* = 0.461, r = 0.198) and Readiness to lose weight (*Z* = −1.19, *p* = 0.236, r = 0.318) among the whole sample. Indeed, median RR score was 8.0 both at T0 and T1 for the Readiness to exercise more and Readiness to lose weight variables. When we split the sample into 2 groups (EG, CG), differences between T0 and T1 in RR were still no significant [EG: Readiness to exercise more (*Z* = 0.00, *p* = 1.000, r = 0), Readiness to lose weight (*Z* = −0.82, *p* = 0.414, r = 0.290); CG: Readiness to exercise more (*Z* = −1.00, *p* = 0.317, r = 0.408), and Readiness to lose weight (*Z* = −0.82, *p* = 0.414, r = 0.335)].

### Usability and satisfaction

Participants’ scores on the SEQ revealed a good usability of the virtual platform (*M* = 51.31; *SD* = 13.38), while a Mann–Whitney U test showed that there were no significant differences between the EG and the CG on the SEQ scores [*U*(*N*_EG_ = 8, *N*_CG_ = 6,) = 22.000, *Z* = −0.26, *p* = 0.795, r = 0.069]. Regarding participants’ responses on individual SEQ items, the only significant difference with large effect was for item 11 (*“¿Do you think this treatment will be useful for you?”*) [*U* (*N*_EG_ = 8, *N*_CG_ = 6) = 3.500, *Z* = −2.83, *p* = 0.005, r = 0.756], on which higher scores were found among participants from the EG (*Mdn* = 5) compared to the CG (*Mdn* = 3).

### Body ownership

**Figure 1:**
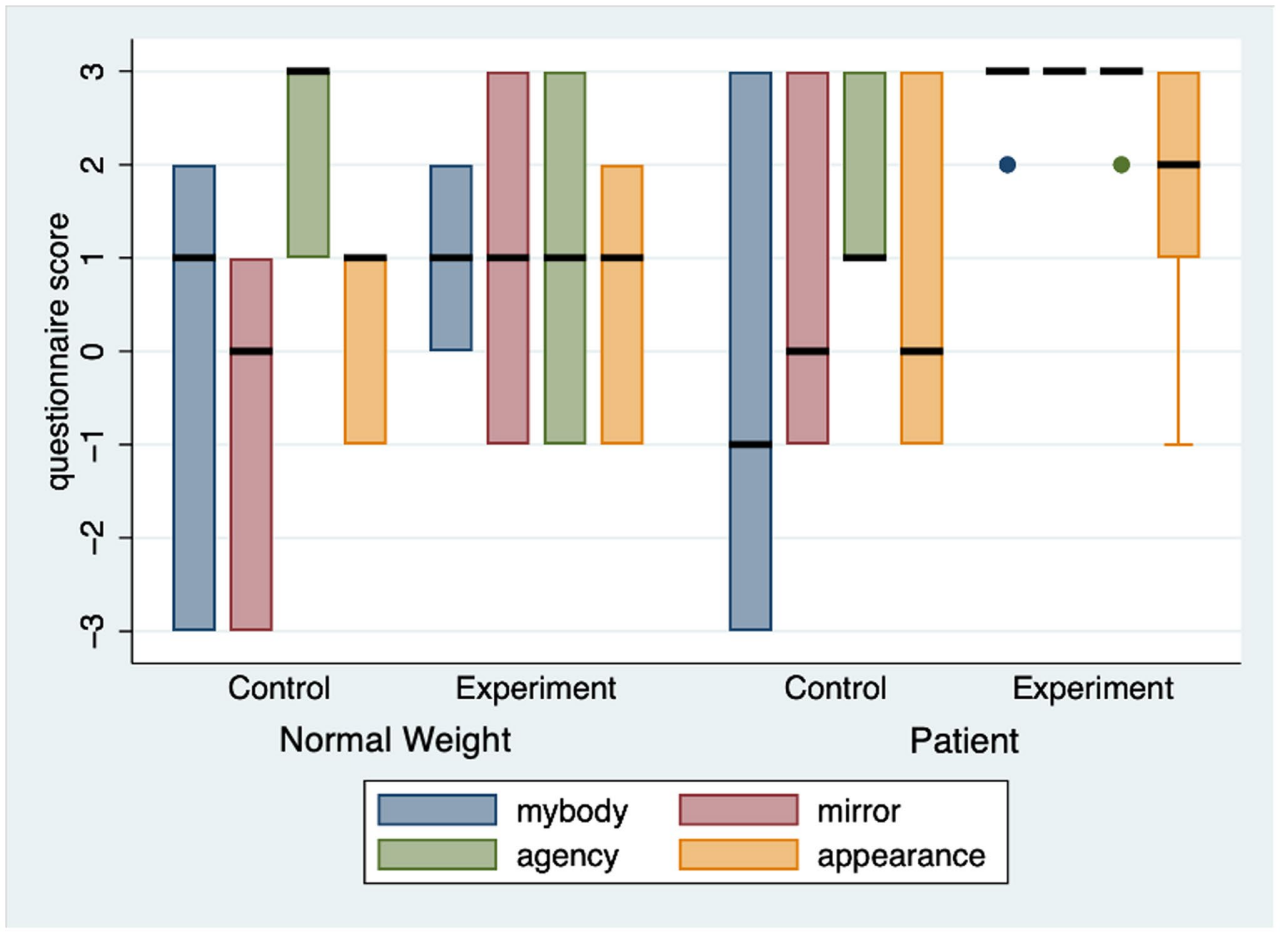
Item scores from Body ownership questionnaire comparing Control and Experimental group, separately distributed in normal weight participants and patients.

Non-statistically significant differences in the BOQ scores were shown between treatment conditions, except for the “body ownership-mirror” item (*“Even though the body I see might not physically look like me, I feel that the virtual body I see reflected in the mirror is my body”*), in which the EG (*Mdn* = 3) reported higher body ownership when looking at the mirror than the CG (*Mdn* = 0) [U(*N*_EG_ = 8, *N*_CG_ = 6) = 9.000, *Z* = −2.07, *p* = 0.038, r = 0.553]. Figure 1 shows BOQ scores between EG and CG, separately for normal weight participants and PLWO.

### Sex as a covariate

One-Way ANOVA with Bootstrapping (1,000 samples) with Sex as covariate was performed for all tests when comparing between EG and CG. The only deviation from the previously reported results was found for “body ownership-mirror” item of the BOQ, in which, in opposition to previous analysis, no significant results were reported between groups [*F*(1, 11) = 2.44, *p* = 0.146, η^2^ = 0.182].

### SEQ-BMI correlation

**Figure 2:**
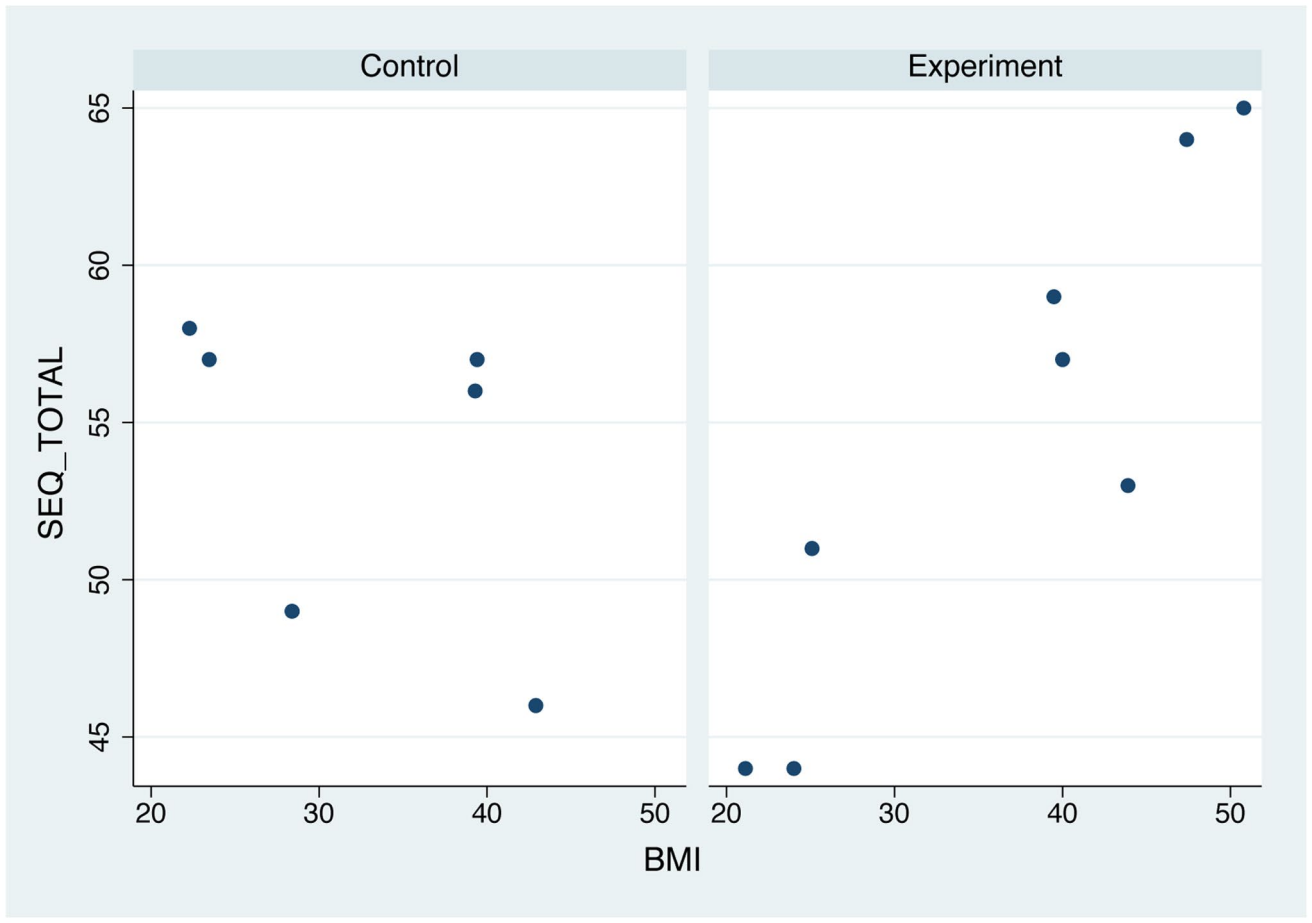
Relationship between body mass index and suitability evaluation questionnaire scores separately distributed in normal weight participants and patients.

As shown in Figure 2, Spearman’s correlations showed a strong positive correlation between the SEQ Total score and the BMI [*r*(6) =0.916, *p* = 0.001] for participants from the EG, while this correlation was no significant for the CG [*r*(4) = − 0.750, *p* = 0.086].

### Qualitative results

The qualitative analysis focused on results from the interviews carried out with participants from the EG and the CG right after their virtual experience (T1). Participants´ responses were categorized into different key themes that were then classified into different domains, following previous research on mHealth adoption barriers and facilitators carried out by a member of the research team (32). The list of domains and themes together with the perceived advantages and disadvantages of the platform for the EG only is shown in Table 3. Finally, the perceived advantages and disadvantages of the virtual experience perceived by participants of the CG will be reported in the last part of this section.

For the EG, regarding the advantages of the *“Perceived usefulness”* theme, all participants (8/8) agreed that the VR platform enhanced their motivation to change regardless of their health condition (i.e., “*It makes you think and say to yourself: Wow, this is something I have to do*,” “*It helped me a lot because I realized that my problem has a solution*”). Also, 6/8 participants considered *ConVRSelf* as a new source that helped them to understand their reasons for change and express them through the “Change Talk” (6) (i.e., “*It helped me to reflect and be more aware of the steps that I must follow to achieve my goals in a more concrete and tangible way,*” *“It confronts you with your goals and helps you to better organize your ideas”*). Besides, all participants (8/8) considered that *ConVRSelf* enriched their perspective on the problem and gave them more self-confidence (i.e., *“Sometimes, it is hard to listen to the others’ advice, and we tend to trust our own beliefs more. Contrary to this, this platform is very useful because it helps you listen to your own advices as if these were given from the outside”; “I have been able to immerse myself into the virtual experience, gain confidence, and not feel ashamed when explaining my problem to someone I did not know”*).

Regarding the disadvantages of the usefulness theme (see *“Negative perception of usefulness,”* Table 3), 2 normal weight participants agreed on the fact that changing perspectives (body swapping) may be confusing and distracting in the short-term (i.e., *“…I felt disconnected and lost fluency in my argumentation,” “During the self-conversation, I felt disconnected and a bit stressed with the change of roles*”; *“being patient or counselor… I was lost and I did not know if I had to talk as a patient or counselor”*) and 1 highlighted the importance of receiving more previous training on the body swapping technique (*“To use it properly, I consider it essential to receive previous training on the correct use of the platform and also on how to correctly formulate questions for the self-conversation”*).

Apart from being useful, 3/8 participants claimed that the platform was intuitive and easy to use from the beginning (see “*Perceived Ease of Use,”* Table 3) and the rest claimed that managing the platform was not easy until becoming familiarized with it (i.e., *“At the beginning I needed some instructions and guidance from the therapist but then it was easy for me to use the platform*”). As regards the *“Negative perception of ease of use,”* some participants (5/8) showed discomfort when using the controllers at the beginning of the experience highlighting the need to improve their sensitivity (i.e., *“I would improve the sensitivity of the controllers, as it was difficult for me to use them properly and to press the buttons that I was asked to continue with the experience correctly*”). Besides, a left-handed participant also mentioned that the platform was not well-adapted to her needs (i.e., *“Being left-handed was quite challenging because the platform thought I wanted to push the Exit button when I was just rising my right arm”*).

As regards the participants’ perceived advantages concerning the design of the platform (see “*Design and Personalization*,” Table 3), most participants (7/8) considered that the virtual environment was relaxing, calm, and attractive. They also agreed upon the fact that an appealing environment helped them to better communicate with their counselor and concentrate on the task (i.e., “*I found the virtual environment attractive and cosy, and the fact that it was empty was relaxing and promoted my concentration*”). Remarkably, 3/5 of PLWO valued positively the resemblance of the embodied virtual avatar with their real self (i.e., “*The avatar was very similar to me, it wore the same shirt, and the face was as small as mine*”). As regards participants’ perceived disadvantages in relation to the platform’s design and personalization (see *“Problems with the design of the platform and Lack of personalization,”* Table 3), 1/3 of normal weight participants found the virtual environment quite unrealistic for being a counselor’s office (i.e., *“I would prefer a more realistic virtual environment”*). Besides, 1/3 of normal weight participants and 1/5 of PLWO did not recognize themselves in the avatar they embodied (*“The look-alike avatar did not look like me; it was fatter than me, and this was annoying during the experience”*).

As regards the participants’ perceived disadvantages related to the ease of use of the Meta Quest 2 device (summarized in the “*Characteristics of the Oculus Quest 2”* domain, Table 3), all normal weight participants (3/8) identified some disadvantages related to the weight of the Head-Mounted display, concerns related to the proper disinfection of the device, and some discomfort felt in their eyes after the experiment. Finally, a normal weight participant (1/3) suggested that the platform is not adapted to the needs of the elderly and expressed her concerns regarding the digital gap for them (see *“Individual factors”* domain).

Lastly, regarding the CG, the main advantages reported were having an enjoyable experience, which made them increase the awareness of their condition and verbalize their concerns (*Normal weight: “It made me more aware of my habits”; PLWO: “Verbalizing my thoughts was relieving and made me increase my awareness of my condition”*), the ease of use of the platform (*PLWO: “It was very easy to use”*), the attractiveness of the virtual environment, and the empathetic attitude of the counselor (*Normal weight: “What I enjoyed the most was the environment, in particular the sky, and the counselor sympathy”*). On the other hand, the most recurrent criticism was the lack of personalization of the virtual environment and the lack of the opportunity to interact with the counselor (*Normal weight: “The platform was very static and not very personalized”; PLWO: “I missed a more personalized experience that would allow me to have a more intuitive dialogue with my therapist”*).

## Discussion

The objective of the present study was to examine the usability of *ConVRSelf*, a VR platform that has been specifically designed to address some of the psychological needs of PLWO, by using embodiment and body swapping techniques. One of the main challenges for experts working in the area of eHealth is to make the technologies they use friendly, satisfying, and useful for individuals (33). Focusing on the group of patients with obesity and the use of VR for their treatment, several studies have studied the acceptability of VR platforms for this population (34–39) as well as the efficacy of VR modules as complementary tools to standard face-to-face treatment (40, 41). However, what those studies normally offer is an extension of face-to-face CBT techniques for use in a VR context [for instance, behavioral modeling (36), food exposure with response prevention, distraction (39), or exposure to negative body-related experiences (40, 41)]. Some of the psychological factors associated with obesity, such as the ambivalence to make lifestyle changes or the internalization of weight bias, have not been properly addressed in previous studies using VR platforms. In contrast, our study focused on the specific psychological needs of this population in relation to the new technology they were expected to use, and explored their perceived facilitators or barriers to using the VR platform, or their own suggestions for improvement. The long-term aim of the SOCRATES project is to evaluate the efficacy of *ConVRSelf* for PLWO, through a RCT, after having carried out the present usability study.

Participants from both groups showed high readiness to change lifestyles before engaging with the virtual experience, which was maintained at the same level after the intervention. It is interesting to note that PLWO were already attending the Endocrinology and Nutrition Department of the Vall d’Hebron University Hospital to seek treatment, something that may explain why they showed high motivation to engage with a healthier lifestyle even before the virtual experiment. This remarkable finding should be explored in more detail in the ongoing RCT with a larger sample size and longer follow-up assessments. In addition, to say that someone is ready to change is not the same as being confident about change, wanting to change, or making a commitment to change. In the RCT, these multifaceted elements of motivation will be differentiated and measured separately. On the other hand, all participants revealed good usability of the platform, with no significant differences in the total SEQ scores between the EG—with the body swapping element—and the CG—without body swapping. Similarly, in a previous study using *ConVRSelf* with healthy volunteers, it was found that the virtual experience was associated with positive psychological changes among healthy participants, irrespectively of the presence or not of the body swapping element in their virtual experience (14).

Regarding the BOQ, no differences were found between the two treatment conditions in the embodiment-related questions. As was suggested by Slater and colleagues (14), for the experiment with such conditions to be optimal, strong illusions of body ownership are necessary, and ought not to differ between the conditions. This seems to have been achieved with our experiments in which we compared BOQ differences between groups controlling for sex as covariate.

Finally, there was a strong association between BMI and usability results among people from the EG. This result is optimistic as it shows that the *ConVRSelf* platform with both the embodiment and body swapping elements has been satisfactorily adapted to address the specific needs of people with higher BMI. Also, this finding is relevant for the future RCT and justifies our decision to broader the BMI limit of the inclusion criteria to include also patients with “severe” obesity (BMI > 45).

Regarding the qualitative information obtained from the interviews, almost all participants found the VR experience to be novel, interesting, and enjoyable, with higher acceptability among PLWO from the EG than normal weight participants from the same group. In particular, people from the EG who engaged in the self-conversation with *ConVRSelf* achieved a better understanding of their condition and the associated problems, and through the motivational self-conversation, they managed to reflect on the next steps they should follow to achieve their goals. This response from the EG suggests that the MI training had been fruitful and that participants were able to learn how to use MI techniques and to practice these using the virtual platform to increase their motivation to enhance with healthy lifestyle habits.

As regards the design of the platform, almost all participants revealed high satisfaction with the design of the platform, finding it attractive, realistic, and relaxing. Most PLWO reported satisfaction with their look-alike avatar while one normal weight participant and one PLWO criticized the avatar as being unrealistic.

Some difficulties in using the VR tool were observed, but it is worth highlighting that most of these issues are easily overcome with further training of the users on MI and the correct use of the platform, and minor adjustments in the platform. No privacy and security issues were reported, and neither was any difficulty related to the sex or educational level of the participants. Regarding the age factor and according to a participant’s statement, for older participants, learning to use these technologies could be harder. Little incidence of simulator sickness was reported, and no further emotional and mental issues occurred during the virtual experiences.

As regards the limitations of the study, first, the sample size was small, something that decreases the statistical power of our findings, and participants were not evenly distributed among groups. Due to this reason, intragroup analyses exploring differences between normal weight participants and PLWO in the dependent variables were not performed, whose results should be additionally controlled for those variables that were found to differ significantly between groups (for instance, age and educational level). Given the intervention delivered to the EG, we consider that for theoretical reasons the MI training cannot be separated from virtual self-conversation. The differences between groups should be interpreted with caution since the observed effect will always be shared and not reduced to a single factor (MI training or virtual self-conversation).

Finally, regarding future improvements in the platform to address some of the reported difficulties, first, we are willing to offer patients more options to personalize their virtual environments (i.e., by selecting the counselor’s avatar, including young normal weight, old normal weight, young overweight, and old overweight). Second, some participants were not satisfied with their look-alike avatar and criticized how little resemblance they bore to their real selves. To address this limitation, we intend to improve the voices of the avatars and to validate the resemblance of each avatar by a researcher of the SOCRATES team, before exposing patients to the virtual experience. Thirdly, previous training on the correct use of the platform will be carried out through a video presentation of how to use *ConVRSelf*. This way, participants will have more tools to be autonomous during the experience. Fourth, to make the virtual environment more relaxing, we have incorporated some elements reminiscent of a therapist’s office (i.e., a picture of Sigmund Freud behind the avatar of the virtual counselor). Fifth, to avoid role confusion in the body swapping experiments, we are planning to add some simple elements to make participants better differentiate their embodiment in each one of the two virtual perspectives (i.e., deeper counselor’s voice, and different items behind each virtual body). Finally, the research team has incorporated a Clean Box^1^ for disinfection of the Quest 2 device, which is based on UVC light sanitation.

The present usability study has provided an important advance in the development of the SOCRATES project and in particular, the preparation of the RCT. It has shown that the *ConVRSelf* system is well-accepted by participants and is now ready to be tested with PLWO in a clinical setting. Respectively, through the upcoming RCT with ambulatory patients with obesity recruited from the Vall d’Hebron University Hospital, we expect to demonstrate the effectiveness of the motivational self-conversation in VR using embodiment and body swapping techniques in improving several psychological outcomes of these patients, such as several elements related to motivation to change habits (importance, confidence, and readiness) as well as psychological wellbeing. Finally, through the integration of MI in the VR context with the patient being properly trained to carry out his/her own motivational self-conversation, we will provide an important advance in the psychological treatments for obesity by addressing some of the root psychological factors associated with the problem and promote patient-centered interventions for this population.

## Data availability statement

The raw data supporting the conclusions of this article will be made available by the authors, without undue reservation.

## Ethics statement

The current study, which involved human participants, was reviewed and approved by Drug Research Ethics Committee and Research Projects Committee of the Vall d’Hebron University Hospital. The patients/participants provided their written informed consent to participate in this study.

## Author contributions

DA, MS, and PLP conceived the study. DA, JVD, ARQ, AC, MC, and PLP were responsible for the clinical implementation of the study while BS, EAD, and MS adapted the virtual platform to the needs of people with obesity and offered technical support during the study. The whole team developed the study design, DA wrote the Introduction, and along with PGR and PH, they wrote the Methods, the Results, and the Discussion sections of the manuscript. All authors contributed to the refinement of the manuscript, reviewed it, and approved its final version. Authorship eligibility for this publication was determined in accordance with the European Code of Conduct for Research Integrity (https://allea.org/code-of-conduct/#toggle-id-3).

## Funding

This study has been funded by the European Union’s Horizon 2020 Research and Innovation Programme (grant agreement no 951930). The funders had no role in study design, data collection and analysis, decision to publish, or preparation of the manuscript. This study was also financially supported by the Serra Húnter program in the form of a grant awarded to DA.

## Acknowledgment

We would like to thank the rest of the members of the SOCRATES consortium for their contributions to the project.

## Conflict of interest

MS and BS are Founders of the University spin-off company Virtual Bodyworks. EÁ was employed by Virtual Bodyworks S.L.

The remaining authors declare that the research was conducted in the absence of any commercial or financial relationships that could be construed as a potential conflict of interest.

## Publisher’s note

All claims expressed in this article are solely those of the authors and do not necessarily represent those of their affiliated organizations, or those of the publisher, the editors and the reviewers. Any product that may be evaluated in this article, or claim that may be made by its manufacturer, is not guaranteed or endorsed by the publisher.

## Footnotes

^1^https://cleanboxtech.com/
